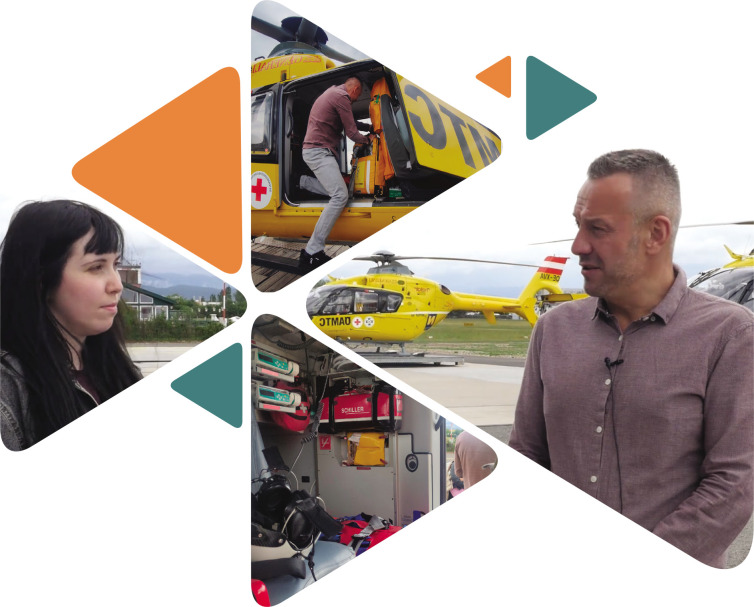# Daniel Csomor – Adapted interviews from Neurotrauma Treatment Simulation Center (NTSC) – Vienna, 2022

**DOI:** 10.25122/jml-2022-1022

**Published:** 2022-07

**Authors:** Alexandra-Mihaela Gherman, Diana Chira, Andreea Strilciuc

**Affiliations:** 1. RoNeuro Institute for Neurological Research and Diagnostic, Cluj-Napoca, Romania

**Interviewee: CSOMOR Daniel**

**Interviewer: Diana CHIRA**

**D.C**.: Can you please tell us a few words about yourself, like your focus area in practice?

**C.D**.: My name is Csomor Daniel, I am specialised in anaesthesia in the intensive care unit, and my focus area is internal care medicine.I am working in the General Hospital in Vienna Neustadt, and the second focus area which I also love is pre-hospital emergency medicine.

**D.C**.: What motivated you to be part of this program, today?

**C.D**.: To share expertise is really nice, not only to students which we sometimes teach, [...] not only to young colleagues but [...] also colleagues coming to Vienna Neustadt who are experienced [...] To share expertise, this was the motivation to participate.

**D.C**.: O.K. Tell me how your experience here shaped your opinion regarding the treatment of neurotrauma.

**C.D**.: For 15 years I am regularly on duty on the emergency medical helicopter so there are many experiences I had.

So, not every duty means you will deal with traumatic brain injury, it depends on the pre-alert system, and what patients need my assistance, but in general one can say, every day, every other day, I am dealing with a traumatic patient, with an injured patient.

**D.C**.: So you do encounter a lot of neurotrauma.

**C.D**.: Yes, I think so.

**D.C**.: And regarding our program, are you familiar with similar programs like this one, at the moment, or are you involved in any of them? You mentioned a congress…

**C.D**.: I have to say, there is a congress, but that's something different. It's the international congress of experts on air medicine. That's where I'm going to participate in June. But scholarships or programs like NTSC (Neurotrauma Treatment Simulation Center), I was not involved in beforehand.

**D.C**.: Can you tell me some challenges in the approach to TBI at the moment? Do you have any idea what we could do to prevent neurotrauma?

**C.D**.: That is a different question because in medicine how we do it pre-hospital, in the shock room and intensive care station, there is nothing really to improve, I would say [...]. But to prevent… this is primarily, the prevention, how to prevent the traumatic brain injury, but that's not only a job for the doctors, that's also the job of the politics.

**Figure:**
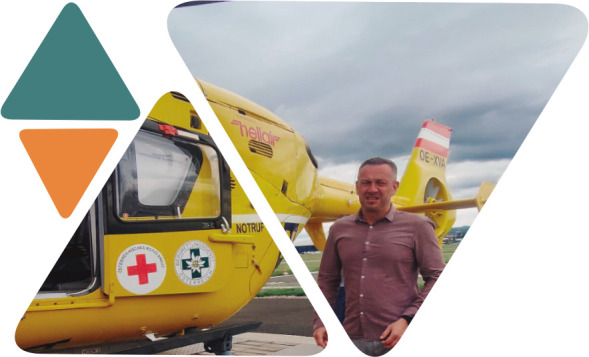


**D.C**.: Yes, that's right.

**C.D**.: Wearing a helmet during skiing, for example, is a standard in Austria, I have to say it.

**D.C**.: And about the cases that you've encountered, could you tell us a few words about, maybe the most complex and difficult case in your career, so far?

**C.D**.: When a patient comes to my intensive care department being critically ill [...] so there is one vital function which doesn't work, [...] that's the complexity. One can not make therapy only for a liver, for example, or heart or brain.

Every person [...], no matter the reason for coming to the intensive care department, is critical. [...] I can tell you… [...] behind you see the big wall, the alpine setting, there was a family who dropped down 150 metres, the father died in this accident, one child died and the second child, we managed to rescue, with a good neurological outcome. That's maybe a case which I think a lot about.

**D.C**.: I see. Online communities in the medical field are becoming more and more visible. Considering this, do you think is it a good thing, like an online community would help medical practice today?

**C.D**.: Yes, of course. I think everybody who can help to prevent something or do something, it's important. It's important for the patient.

**D.C**.: Do you consider that Covid-19 has impacted the management of neurotrauma and how? And maybe your work overall?

**C.D**.: Yes, of course. There was an impact of this pandemic, definitely. First of all, the people stayed at home, so we saw fewer injured people during this time.

**D.C**.: So it affected basically also in a good way.

**C.D**.: No, that's not a good way, because the intensive care department was full of Covid ill patients, so when something happened, we had to search [...] for a free opportunity where we can bring the patient because the intensive care unit was full of Covid patients.

**D.C**.: We had one more question, but I guess the noise [from the helicopters] will have to…

[The dialogue is interrupted by the noise of helicopters taking off and preparing for intervention missions. The first one, as C.D. explains, with a mission in upper Austria, in Linz, and the second one, down to a village behind the hill, where a patient awaits rescue.]

**D.C**.: O.K. So, our last question was, from your perspective, which are the aspects that would affect the proper long-term follow-up of patients?

**C.D**.: That is a difficult question for me, because that's not my everyday job, to follow up with the patient. Unfortunately, I lose my patient in the follow-up, when the patient is transferred to a normal ward, to neurology or neurosurgery or traumatology ward.

And then, after leaving the hospital for rehabilitation, it is seldom that patients come back to us, to the intensive care unit and ask 'Do you know me?' [...] So many patients look different and [...] we don't recognize them, unfortunately.

And that's the only follow-up I sometimes have, but it depends on the patient himself to come and say 'Thank you!' or something like that. So in my job, in intensive care medicine, I have no follow-up of the patients.

**D.C**.: [...] Thank you for your time, and thank you for your presentations!

**C.D**.: Thank you very much!

Watch the extended interview on the AMN Website: https://brain-amn.org/ntsc-interviews-daniel-csomor/

**Figure:**